# Proceedings of the 2013 MidSouth Computational Biology and Bioinformatics Society (MCBIOS) Conference

**DOI:** 10.1186/1471-2105-14-S14-S1

**Published:** 2013-10-09

**Authors:** Jonathan D Wren, Mikhail G Dozmorov, Dennis Burian, Rakesh Kaundal, Andy Perkins, Ed Perkins, Doris M Kupfer, Gordon K Springer

**Affiliations:** 1 Arthritis and Immunology Research Program, Oklahoma Medical Research Foundation; 825 N.E. 13th Street, Oklahoma City, OK 73104-5005, USA; 2 Biochemistry and Molecular Biology Dept, Univ of Okla Health Sciences Center, USA; 3 Civil Aerospace Medical Institute, Federal Aviation Administration, Oklahoma City, OK 73169, USA; 4 National Institute for Microbial Forensics & Food and Agricultural Biosecurity, Department of Biochemistry & Molecular Biology, Oklahoma State University, Stillwater, OK 74078, USA; 5 Department of Computer Science and Engineering, Mississippi State University, Mississippi State, MS 39762, USA; 6 US Army Engineering Research and Development Center, Vicksburg, MS 39180, USA; 7 Computer Science Department, University of Missouri, Columbia MO, USA; 8 University of Missouri Bioinformatics Consortium, USA

**Keywords:** bioinformatics, conferences, MCBIOS, ISCB

## Introduction

The tenth annual conference of the MidSouth Computational Biology and Bioinformatics Society (MCBIOS 2013), "The 10th Anniversary in a Decade of Change: Discovery in a Sea of Data", took place at the Stoney Creek Inn & Conference Center in Columbia, Missouri on April 5-6, 2013. This year's Conference Chairs were Gordon Springer and Chi-Ren Shyu from the University of Missouri and Edward Perkins from the US Army Corps of Engineers Engineering Research and Development Center, who is also the current MCBIOS President (2012-3). There were 151 registrants and a total of 111 abstracts (51 oral presentations and 60 poster session abstracts).

Keynote speakers were John Quackenbush from the Dana-Farber Cancer Institute, whose talk was titled "The Road to Genomic Medicine is Paved with Data", and Veronica Vieland from Nationwide Children's Hospital and The Ohio State University, who spoke on "Is the Universe Made of Information?" Dr. William Slikker, Director of the Food and Drug Administration's, National Center for Toxicological Research, concluded with a talk on the past ten years of MCBIOS and a perspective on its future.

Participants also had the opportunity to attend several workshops: 1) Dr. Peter Cooper, National Center for Biotechnology Information/NIH, NCBI Tools Webinar. 2) Dr. Wieda Tong, National Center for Toxicological Research/FDA, ArrayTrack Workshop. 3) Drs. Joshi Trupti & Dong Xu - University of Missouri, Soybean Knowledge Base (SoyKB): A Powerhouse for Soybean Research and Breeding Workshop

The winners of conference awards were:

**Best Paper Award**: Mikhail G. Dozmorov *et al. *"Systematic classification of non-coding RNAs by epigenomic similarity" [1]

Best Paper Runner-up: Yi Yang *et al. *"Differential Reconstructed Gene Interaction Networks for Deriving Toxicity Threshold in Chemical Risk Assessment" [2]

Best Oral Presentations (Post-Doctoral fellows):

1st Place: Zheng Wang, University of Missouri

2nd Place: Binsheng Hong, FDA/NCTR

Best Oral Presentations (students):

1st Place: Awantika Singh, UALR/UAMS

2nd Place: Anna Bennet, University of Missouri

3rd Place: Yifei Xu, Mississippi State University

Best Poster (Computation):

1st Place: Tian Gui, University of Mississippi

2nd Place: Surya Kilaparty, UALR

3rd Place: Tamer Aldwairi, Mississippi State University

Best Poster (Biology):

1st Place: Trupti Joshi, University of Missouri

2nd Place: Joseph Reddy, Mississippi State University

3rd Place: Nathan Crabtree, UALR

## Important new changes to the MCBIOS Proceedings

**Figure 1:**
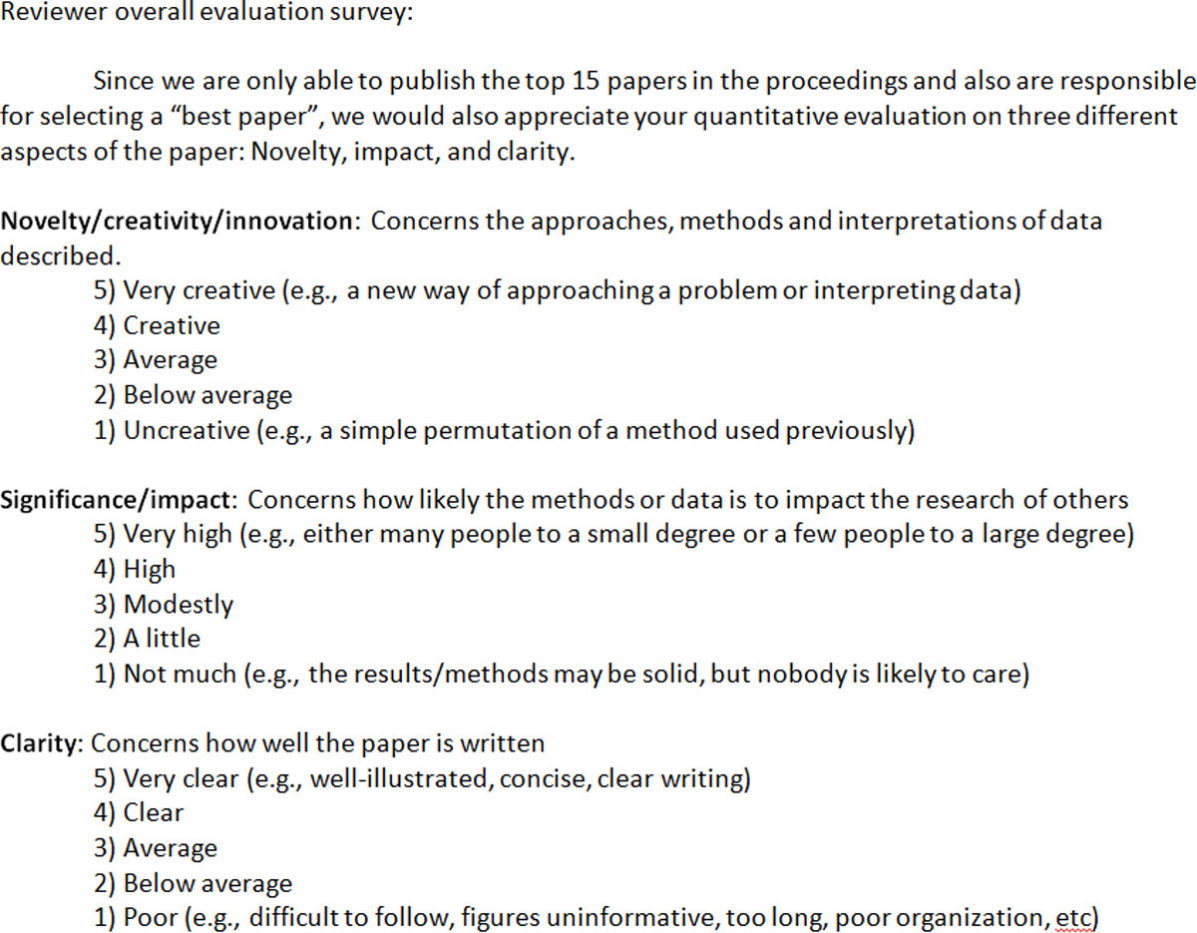


This year, BMC requested that MCBIOS limit the number of papers published in the Proceedings to the top 15, which necessitated the introduction of a new system to rank papers. The downside is that it changed our long-standing goal of being as inclusive as possible to accommodate all papers judged scientifically sound and relevant by the reviewers, to one where not all worthy papers could be accommodated. The upside is that it enabled us, for the first time, to select a "Best Paper" award. To determine this, all papers deemed acceptable by reviewers were quantitatively evaluated on the basis of three criteria: Novelty, Impact and Clarity (reviewer questionnaire shown in Figure 1). This enabled the papers to be ranked. The top 3 papers were then sent to the Board of Directors to either approve the final ranking of papers or raise concerns if any (e.g., if reviewer scores in a particular category were very divergent). It was also the Board's responsibility to resolve ties (when applicable) by a democratic and public vote. Editors who were also co-authors of submitted papers were not permitted to handle their own papers editorially. Similarly, Board members who were authors on a paper under consideration for an award, were not eligible to vote.

### Proceedings summary

This year, 27 papers were submitted, 19 papers were deemed acceptable by reviewers, and we were able to accommodate the top 16 (there was a 5-way tie for 16^th ^place), giving an acceptance rate of 59%. The acceptance rate was slightly lower than last year's rate of 65% for the 13 papers accepted in 2012 [3]. All papers were peer-reviewed by two or more reviewers. Papers generally fell into four categories:

### Networks and pathways

Yi Yang *et al*., received the Best Paper Runner-up Award for their study of pathway alterations using a differential network approach (DN) [2]. Their goal was to understand toxicological modes of action and assess biological risk for toxic materials. Their DN approach consisted of 6 steps: time-series gene expression data collection, identification of altered genes, gene interaction network reconstruction, differential edge inference, mapping of genes with differential edges to pathways, and establishment of causal relationships between chemical concentration and perturbed pathways.

Jialiang Yang *et al. *describe an approach to align biological networks called BinAligner [4], which differs from prior approaches in that it attempts to combine global and local alignments. They compare it with other alignment approaches and show its efficiency on alignment of viral protein interactions.

### Transcriptomics

Mikhail Dozmorov *et al. *received the Best Paper Award for their study that involved a method of exploratory genomic analysis to identify statistical enrichments and depletions between non-coding RNA classes and epigenomic features such as histone marks and transcription factor binding sites (TFBS) [1]. Using an application they previously developed, called GenomeRunner, to perform the analysis and comparisons, the authors successfully demonstrate a novel approach to interpreting the significance of co-localization of ncRNA with regulatory epigenetic elements in terms of possible functions and mechanisms by which ncRNA classes are regulated.

Researchers interested in comparing their RNA sequencing data with a variety of public microarray data will note a paper by Shweta Chavan *et al. *The method not only compares the expression of genes of interest between RNA-seq and microarrays, but also monitors the abundance of different isoforms. Wrapped around commonly used tools, this paper demonstrates a viable strategy to efficiently utilize volumes of microarray data in conjunction with modern sequencing technologies [5].

Fan Zhang and Renee Drabier present SASD, an alternative splicing database that can be used to identify isoforms from proteomic studies [6]. It enables an efficient way to identify isoforms such as skipped exons and retained introns from mass spectrometry data.

Cory Giles *et al. *describe MirCoX, a database of miRNA-mRNA expression correlations from a meta-analysis of publicly-available RNA-Seq data [7]. They also found that gene-miRNA pairs identified by tools such as TargetScan and miRanda tended to have anti-correlated expression patterns in the data sets analyzed. A web tool has been developed to query miRNA-mRNA correlations.

Firoz Ahmed *et al. *use support vector machines to predict Dicer cleavage sites [8]. The method, which can make use of structural information, was trained and tested on experimentally-validated miRNAs from miRBase and found to have over 86% accuracy. A web server, PHDcleav, is available to allow users to predict Dicer cleavage sites in pre-miRNA.

### Protein structure

Jie Shen *et al *described three-dimensional modeling of the rat α-Fetoprotein, a plasma protein that can sequester estrogens providing an important buffer in preventing estrogenic compounds from entering target cells [9]. A 3-D model of α-Fetoprotein was built using the structure of rabbit serum albumin as a template, which was then optimized using Molecular Dynamic simulation. The model was then used to examine binding of 13 different α-Fetoprotein binders leading to the identification of key binding residues in the α-Fetoprotein. This model will be useful in estimating binding affinities of chemicals to α-Fetoprotein in the evaluation of the endocrine disruption potential of chemicals.

Eickholt and Cheng [10] addressed the problem of predicting inter-residue contacts in protein structures, which has premier applications in protein folding problem, structure-based drug designing, *etc*. The authors conduct a comparison of programs that participated in CASP10, including their own method DNcon, for their efficiency at predicting residue-residue contacts. DNcon, which is a deep network-based method, consistently performed well based on various evaluation criteria, such as L/10, L/5, medium and long rang contact predictions. Their findings explain that how predicted residue-residue contacts have been useful in tertiary structure modeling and model quality assessment.

R. Shyama Prasad Rao *et al. *examine the potential for cross-talk among proteins that are reversibly phosphorylated (on their serine, threonine or tyrosine residues) and their methionine residues which can be oxidated [11]. They find that there is a large proportion of known phosphorylation sites among signaling proteins are enriched for methionine residues being present nearby the phosphorylation sites.

Rakesh Kaundal *et al. *discuss the creation of an identification and classification system for plastid proteins [12]. Firstly, they developed Support Vector Machine (SVM) classifiers to distinguish between plastid proteins and non-plastid proteins. Then, the plastid proteins were further classified into subcategories (chloroplast, chromoplast, etioplast, amyloplast, etc.). They do a brief comparison of their SVM performance with the simple homology based PSI-BLAST and measure performance based on different features. Due to a lack of proteomic analysis tools available in the field, this paper provides some interesting insights into how such tools might be further developed to analyze larger classes of proteomic data.

### Miscellaneous

Halil Bisgin *et al. *provide an approach to High Content Screening (HCS) that uses topic modeling to analyze cellular endpoints in toxicity assays. They show the topics identified are in concordance with the results of experimental toxigenomic assays [13].

Rahul Singh *et al. *present an approach for interaction and visualization of large and/or complex datasets, among them a system called XMAS for exploration of microarray data and PSPACE for structure-function analysis [14]

Zhendong Zhao *et al. *present an approach to predict drug activity based upon a machine-learning approach to analyze structural conformers [15]. Computational methods are particularly important in this type of problem due to the time and expense associated with experimental testing of all possible conformers, which can be prohibitively large. They show it has competitive discriminative power on the four datasets analyzed.

The foodborne pathogen *Salmonella enterica *can be rapidly characterized via genotyping and pulsed field gel electrophoresis (PFGE). Large amounts of PFGE data are stored by disease monitoring agencies such as the US Center for Disease Control so that new isolates can be matched to known isolates in order to better assess outbreaks of foodborne illness. In order to quickly access and analyze PFGE fingerprinting, Wen Zhou *et al. *examined data mining tools, for the characterization of Salmonella [16]. Several tools were demonstrated to facilitate PFGE data retrieval, interpretation and serotype identification from a PFGE database. These tools and database could facilitate identification of Salmonella strains as well as tracking back to the sources of outbreaks.

Finally, Jason Hennessey and Xigin Ge conducted a study of website (URL) decay in MEDLINE, a problem which grows in importance as the number of public sites for storage and analysis of scientific data grows. They found that websites continue to decay in a time-dependent manner, and examined how many of them were archived, finding that the archival sites contained only a fraction. They conclude by contributing to URL stability by automating the submission of published URLs to these sites [17].

### Future meetings

The Wes Watkins Center, Oklahoma State University (OSU) in Stillwater, Oklahoma will be the site of XI^th ^MCBIOS conference in 2014 to be held March 6-8, tentatively entitled "*From Genome to Phenome - Connecting the Dots*". The 2013-2014 MCBIOS President is Andy Perkins from Mississippi State University, and board member Rakesh Kaundal from OSU is the Conference Chair. Chaoyang (Joe) Zhang from the University of Southern Mississippi, is now the President-elect. MCBIOS is a regional affiliate of the International Society for Computational Biology (http://www.ISCB.org). For information regarding MCBIOS and our future meetings, see http://www.MCBIOS.org.

## Competing interests

The authors declare that they have no competing interests.

## Authors' contributions

All authors served as editors for these proceedings, with JDW serving as Senior Editor. All authors helped write this editorial.

## Declarations

Funding for publication of this editorial came from the Midsouth Computational Biology and Bioinformatics Society (MCBIOS).

This article has been published as part of *BMC Bioinformatics *Volume 14 Supplement 14, 2013: Proceedings of the Tenth Annual MCBIOS Conference. Discovery in a sea of data. The full contents of the supplement are available online at http://www.biomedcentral.com/bmcbioinformatics/supplements/14/S14.
